# *In vivo* stem cell tracking using scintigraphy in a canine model of DMD

**DOI:** 10.1038/s41598-020-66388-w

**Published:** 2020-06-30

**Authors:** Inès Barthélémy, Jean-Laurent Thibaud, Pauline de Fornel, Marco Cassano, Isabel Punzón, David Mauduit, Jean-Thomas Vilquin, Patrick Devauchelle, Maurilio Sampaolesi, Stéphane Blot

**Affiliations:** 1 Inserm U955-E10, IMRB, Université Paris Est Créteil. Ecole nationale vétérinaire d’Alfort, 94700 Maisons-Alfort, France; 2 MICEN-Vet, 94000 Créteil, France; 3 Translational Cardiomyology Lab, Stem Cell and Embryo Biology, Dept Development & Regeneration, KU Leuven, Leuven, Belgium; 4 Department of Biosciences, University of Milan, via Celoria 26, 20133 Milan, Italy; 5 Sorbonne Université, INSERM, AIM, Centre de Recherche en Myologie, UMRS 974, AP-HP, Hôpital Pitié Salpêtrière, 75013 Paris, France

**Keywords:** Cell delivery, Neuromuscular disease

## Abstract

One of the main challenges in cell therapy for muscle diseases is to efficiently target the muscle. To address this issue and achieve better understanding of *in vivo* cell fate, we evaluated the relevance of a non-invasive cell tracking method in the Golden Retriever Muscular Dystrophy (GRMD) model, a well-recognised model of Duchenne Muscular Dystrophy (DMD). Mesoangioblasts were directly labelled with ^111^In-oxine, and injected through one of the femoral arteries. The scintigraphy images obtained provided the first quantitative mapping of the immediate biodistribution of mesoangioblasts in a large animal model of DMD. The results revealed that cells were trapped by the first capillary filters: the injected limb and the lung. During the days following injection, radioactivity was redistributed to the liver. *In vitro* studies, performed with the same cells prepared for injecting the animal, revealed prominent cell death and ^111^In release. *In vivo*, cell death resulted in ^111^In release into the vasculature that was taken up by the liver, resulting in a non-specific and non-cell-bound radioactive signal. Indirect labelling methods would be an attractive alternative to track cells on the mid- and long-term.

## Introduction

Cell therapy intending to regenerate diseased tissue with healthy stem cells is envisioned for diverse pathological conditions. Several different tissues are currently being targeted in clinical trials using a wide range of cell types. This diversity of situations is, at least in part, responsible for the mixed success of stem cells trials. Some of these trials have yielded very encouraging results with obvious cell engraftment and contribution to the tissue regeneration^1,2^, others have shown functional improvement through paracrine effects rather than through stem cells engraftment^3,4^, and finally some other trials have shown disappointing results^5,6^. In every case, and particularly when the cells are injected systemically, the question of biodistribution and survival of cells is crucial, not only for safety reasons but also to better understand cell behaviour and to optimise tissue targeting.

To this purpose, cell tracking methods have been developed, relying on diverse *in vivo* imaging techniques^7,8^. The most obvious way to visualise cells *in vivo* using non-invasive methods is to label cells directly before transplantation. This can be achieved using radionuclides, pharmacologically designed to be taken up by cells; examples of these radionuclides include ^99^mTc-HMPAO, ^111^In-oxine and ^124^I-HIB for SPECT imaging, ^64^Cu-PTSM and^18^F-FDG or FHB for PET imaging^9–13^. Alternatively, direct labelling with SPIO, Gd-DTPA or ^19^F allows for cell tracking using MRI/MRS^14–16^. The main advantage of these direct labelling methods is that they are easy to perform and that they provide information on cell biodistribution shortly after transplantation^7,8,14^. The disadvantages include the potential cytotoxicity of some labelling agents, as well as the limited period of cell tracking conditioned by the radioactive decay and the dilution of the signal due to possible cell division or fusion^7^. To address these limitations, indirect labelling methods have been developed; these methods are based on genetic modification of cells to make them express a reporter gene suitable for imaging^17–21^. The main disadvantage of indirect labelling methods is that they require genetic modification, a step that complicates the process and could result in undesired cell biological modifications.

Both direct and indirect labelling methods have been extensively used in preclinical and clinical studies, with the aim of reaching various pathological targets, to better understand *in vivo* cell behaviour^7–21^. However, despite the fact that stem cell therapy has been widely explored as a therapeutic option for genetic muscle diseases, only a few studies have focused on *in vivo* myogenic stem cell tracking in small animal models^9,15,19,21^. Among these diseases, Duchenne muscular dystrophy (DMD) is a particularly challenging pathological condition to address with cell therapy, because the entire muscular tissue should be targeted, and the niche availability and chemo-attraction capabilities can vary upon the pathological state of the muscle^9,22,23^. This genetic X-linked disorder is caused by mutations in the dystrophin gene and affects one boy born out of 3600 to 9300^24^. The dystrophin deficiency leads to muscle degeneration, and affected boys suffer from a progressive and generalised muscle weakness leading to permanent wheelchair use in the second decade, and premature death from respiratory or cardiac decompensation during the third or fourth decade of life^25,26^.

Since the DMD muscle primarily degenerates and ultimately lacks regeneration capacities, cell therapy has appeared as a relevant therapeutic option. Initial cell therapy studies focused on transplantation of myoblasts, the “professional muscle-maker” cells^27,28^. However, these cells have poor migratory capacity following intramuscular or intra-arterial injection, limiting their interest in such a generalised muscle wasting disease^29,30^. To overcome this limitation, other types of cells have been proposed, notably mesoangioblasts (MABs), which are pericyte-derived stem cells that combine myogenic potential with migratory properties^31^. The therapeutic interest of MABs was first demonstrated in rodent models of muscular dystrophies^31^, and further confirmed in a more challenging preclinical model; namely the Golden retriever muscular dystrophy dog model (GRMD)^32^. This large-size model suffers from dystrophin-deficiency, and exhibits clinical and histopathological signs that are similar to those observed in human DMD, in sharp contrast to the mdx mouse model that is mildly affected by dystrophin-deficiency. Based on the promising results obtained in mice, MABs have been evaluated in the GRMD model^32^. In this context MABs were shown able to engraft in muscle following intra-arterial delivery, produce dystrophin, and improve muscle pathology and force^32^.

On the downside, the results obtained showed a great heterogeneity among treated dogs and among muscles located downstream of the injection site, suggesting that the migratory behaviour of cells could vary depending on several unidentified factors^32^. Although strategies to improve the migration ability of MABs have been developed^22,33^, further studies are needed in order to understand the factors that influence muscle targeting. In particular, quantification studies to assess homing of MABs to the muscle and to study the overall biodistribution in large animal models, could be useful in order to understand the *in vivo* behaviour of these cells and develop strategies to improve it. On top of this, a phase I-IIa clinical trial on intra-arterial delivery of MABs in DMD patients showed partly disappointing results^6^. The conclusion of this clinical trial was that some of the factors that can be modulated to optimise MAB-based therapies are the administration route, selection of a favorable pathological state of the muscle for transplantation, and the use of concurrent immunosuppressive treatments that possibly have a significant effect on cell migration and homing^6^. Prior to translating potential improvements into the clinic, these concerns should be addressed in a relevant preclinical model, such as the GRMD dog. Moreover, the relevance of this model could be further enhanced by the development of an i*n vivo* MABs tracking method suitable for the GRMD model.

Therefore we decided to develop an *in vivo*, non-invasive stem cell tracking method to quantitatively assess the biodistribution of cells in the days immediately after transplantation. In order to avoid any genetic modification of the MABs, we chose a direct labelling method: ^111^In-oxine labelling method for scintigraphy detection. This radionuclide has a relatively long half-life (2.8 days) allowing for serial imaging in the days following transplantation and has been extensively used in preclinical and clinical studies to track stem cells^9,10,34^. ^111^In-oxine is also used in clinic to diagnose and locate infection foci by labelling the patient’s leukocytes^35–37^. The aim of this work was to assess if the tracking of ^111^In-oxine labelled MABs using scintigraphy can be used as a method to reliably monitor the location of transplanted cells and to compare cell types, in the relevant GRMD dog model of DMD. This study, the first one to report on stem cell tracking in this model, also aimed to provide a first depiction of the MABs biodistribution pattern in GRMD dogs.

## Results

### Labelling procedure and cell viability

The ^111^In-oxine labelling efficiency was variable among experiments, ranging from 49% to 88% as detailed in Table 1. The immediate post-injection viability of cells was also variable among experiment. A mean value of 67% viability (SD 27.6%) was obtained for MABs and values of 80 and 53% viability were obtained for the two myoblasts experiments, respectively (Table 1). Viability was analysed after injection, and whenever there was a time lapse between labelling and injection the viability of the cell preparation tended to be lower, suggesting that mortality of cells could occur rapidly after labelling.

**Table 1:** Summary of the dogs, and data on transplanted cells.

Name of the dog	Status	Sex	Age (months)	Cell type	Labelling efficiency	Cell Viability
Bigoune	WT	F	17.2	^111^In-oxine	—	—
Aorte	WT	F	29.1	^111^In-labelled leukocytes	63%	96%
B’carre	WT	F	6.8	^111^In-labelled MABs	88%	30%
T’artine	WT	F	59.8	^111^In-labelled MABs	81%	96%
Btrave	WT	F	19.6	^111^In-labelled MABs	64%	72%
Cto	GRMD	M	3.8	^111^In-labelled MABs	70%	77%
Eole	GRMD	M	4.6	^111^In-labelled MABs	54%	42%
Extra	GRMD	M	4.3	^111^In-labelled MABs	63%	35%
Enzyme	GRMD	F	5.1	^111^In-labelled MABs	59%	93%
Enigme	GRMD	F	4.9	^111^In-labelled MABs	65%	93%
Ifi	GRMD	F	4.7	^111^In-labelled myoblasts	49%	80%
Itineris	GRMD	F	4.0	^111^In-labelled myoblasts	55%	53%

### Immediate biodistribution

**Figure 1:**
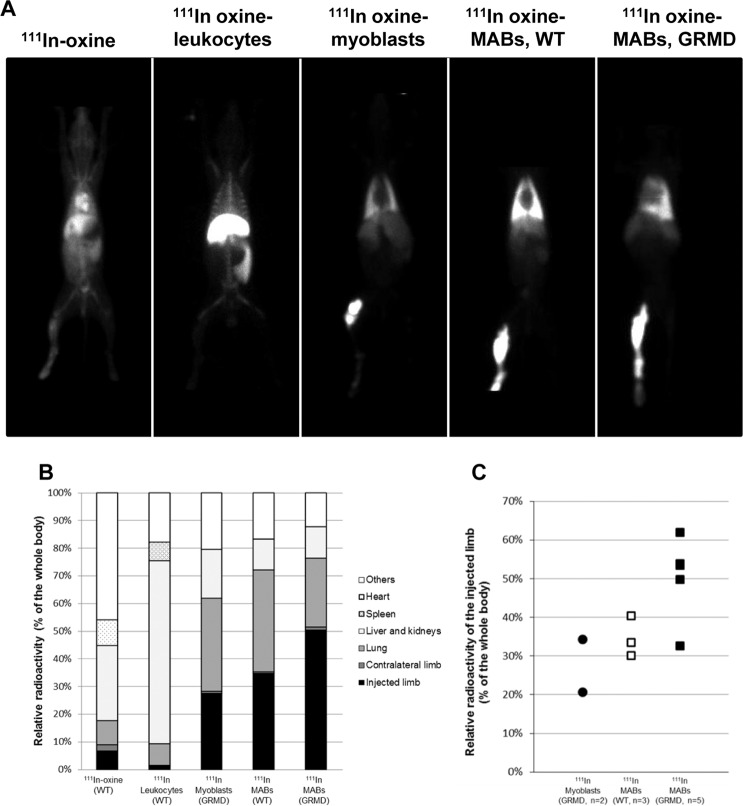
Immediate biodistribution. (**A**) First planar view scintigraphic images acquired 1 hour after intra-arterial injection of either (from left to right) ^111^In-oxine, ^111^In-oxine-labelled leukocytes, myoblasts, or MABs in WT or GRMD dogs. (**B**) The mean proportion of the whole-body radioactivity measured in each ROI on images obtained 1 hour after injection, and for each cell type, is represented as cumulative histograms. The proportion of radioactivity trapped in the injected limb (black) is higher in animals injected with myogenic stem cells (Myoblasts or MABs) and reached the maximal value for MAB-transplanted GRMD dogs. Filter organs (pale grey) trapped a higher proportion of radioactivity in the case of ^111^In-oxine and ^111^In-oxine-labelled leukocytes. (**C**) Individual values of radioactivity measured in the injected limb relative to the whole body for myogenic stem cells. Radioactivity was trapped more efficiently in the injected limbs of MAB-transplanted GRMD dogs, with the exception of one dog that showed relative radioactivity levels that overlapped those observed in WT dogs. The relative radioactivity in the injected limb of myoblast-transplanted GRMD dogs was lower than in MAB-transplanted GRMD dogs, suggesting the trapping of myoblasts in the injected limb in less efficient than for MABs.

The first scintigraphy sequences revealed distinct radioactivity biodistribution patterns for ^111^In-oxine, ^111^In-labelled leukocytes and ^111^In-labelled myogenic stem cells (Fig. 1A). ^111^In-oxine was mainly found in blood and liver, and to a lesser extent in kidneys and skeleton. ^111^In-labelled leukocytes were found in their known homing location: liver, spleen and bone marrow^37,38^. The images obtained following administration of ^111^In-labelled myogenic stem cells (MABs or myoblasts) through the femoral artery revealed that the radioactivity was trapped by the two first capillary barriers encountered: (1) the injected limb and (2) the lung. This capillary barrier effect was seen very clearly in the dynamic acquisitions obtained during cell injection (Figure movie S1).

In one of the GRMD dogs injected with MABs a different biodistribution pattern was observed. This animal showed a marked accumulation of radioactivity in a localised region of the thigh and another weaker spot in the distal part of the limb, but the accumulation in this latter region was smaller than the accumulation observed in the same region of other dogs. SPECT/CT images revealed that medial muscles of the thigh and especially the *vastus medius* muscle were strongly radioactive, suggesting that the injection catheter could have engaged through a collateral artery, perfusing this muscle group specifically (Fig. S2). The other MAB-injected dogs showed a rather homogeneous signal in the injected hindlimb downstream of the catheter. In dogs injected with myoblasts, the signal appeared patchier.

These observations were further confirmed by quantitative analysis of radioactivity (Fig. 1B,C). The proportion of radioactivity of each region versus whole-body radioactivity measured on the first planar static acquisition was assessed. The result of this analysis showed that the relative percentage of radioactivity in the injected limb was below 10% in dogs injected with either ^111^In-oxine or ^111^In-labelled leukocytes while it was above 25% in dogs receiving myogenic stem cells. The relative percentage of radioactivity localised in the MAB-treated limb tended to be higher in GRMD than in healthy dogs (mean 50.4% SD 10.9% vs mean 34.7% SD 5.2%) but this difference was not significant (p = 0.14). In GRMD dogs injected with myoblasts, the mean percentage of radioactivity localised into the injected limb was even lower (mean 27.5%, SD 9.7%), attesting to the ability of this cell tracking method to detect different migratory capacities of myogenic stem cells. Individual data showed that the relative activity measured in the injected limb was variable among dogs; this variability could be attributed, at least in part, to variability in cell viability. Indeed, the two GRMD dogs injected with MABs having the best level of cell viability (93%) were those with the best relative radioactivity level in the injected limb. On the opposite, one of the two GRMD dogs injected with MABs having poor levels of viability (<50%) was the one with the lowest relative radioactivity in the injected limb. In the same way, in the WT group, the dog injected with MABs having the lowest viability level was also the one with the lowest relative radioactivity in the injected limb. The same observation was made in the GRMD dogs injected with myoblasts. A Spearman rank test was performed to assess this correlation in the MAB-injected GRMD group, despite the low number of animals. The R coefficient reached 0.67 but the correlation was not statistically significant (p = 0.22).

The second capillary barrier encountered by myogenic stem cells, i.e. the lung, trapped most of the remaining activity after the first passage through the injected limb. The relative activity measured in the lung was slightly higher than the one in the injected limb in GRMD dogs injected with myoblasts (mean 33.6%, SD 13.6%) and in the WT dogs injected with MABs (mean 36.7%, SD 9.0%). Conversely, in GRMD dogs injected with MABs, the relative activity measured in the lung was half of the one measured in the injected limb (mean 25.0%, SD 13.5%). The difference between GRMD and WT dogs injected with MABs was however not statistically significant (p = 0.14).

### One-week biodistribution follow-up

**Figure 2:**
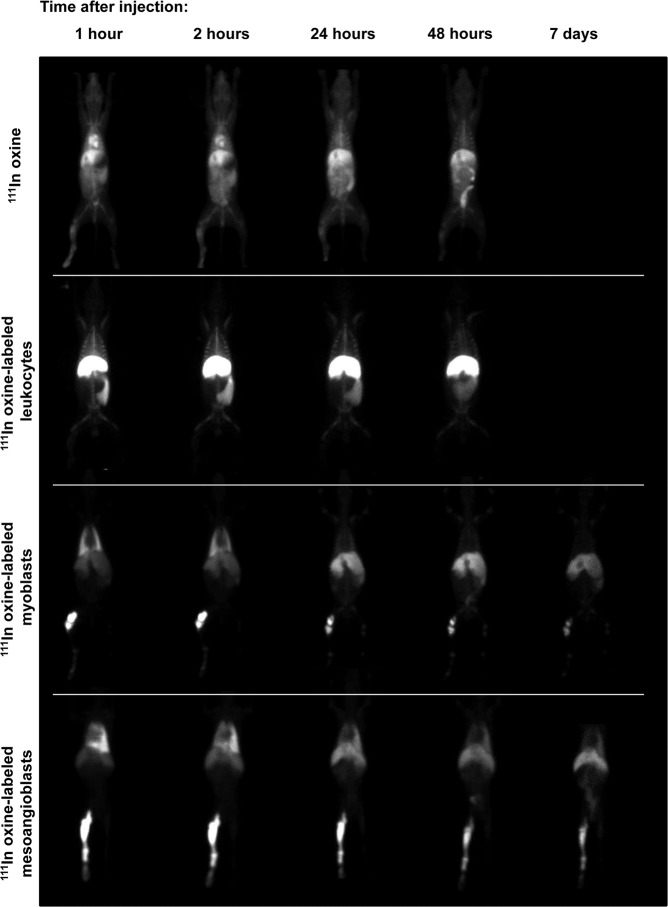
Comparison of whole body scintigraphy images overtime after injection of different ^111^In-oxine vehicles. Representative planar view scintigraphy images obtained at different timepoints (in columns: 1, 2, 24, 48 hours and 7 days) after intra-arterial injection of either ^111^In-oxine (1^st^ row), ^111^In-oxine-labelled leukocytes (2^nd^ row), ^111^In-oxine-labelled myoblasts (3^rd^ row), or ^111^In-oxine-labelled MABs in a GRMD dog (4^th^ row).

Over the one-week follow-up, the images obtained from the ^111^In-oxine and ^111^In-labelled leukocyte-treated animals showed a biodistribution pattern that closely resembled the one obtained on the day of injection. In the case of the myogenic stem cell-treated animals, the radioactivity signal revealed a much more dynamic behaviour. Radioactivity was detected in the treated limb throughout the week after injection, but there was a marked decrease in activity in the lung, where the signal tended to disappear. In contrast, the images obtained during the first week post-treatment indicated that there was a progressive increase in radioactivity in the liver, a tissue where the activity on day 0 was very low (Fig. 2).

**Figure 3:**
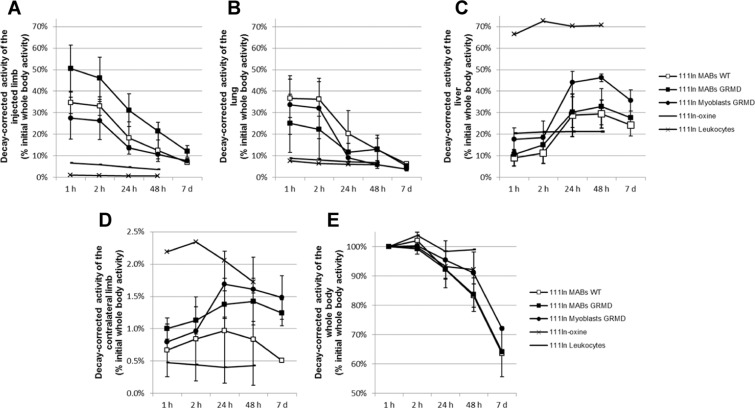
Monitoring of the biodistribution over time. The radioactivity of each of the ROI was expressed as a percentage of the initial whole-body activity, after decay correction, and is represented over time for each of the groups. (**A**) Injected limb. Note the rapid decrease of radioactivity in all dogs injected with myogenic stem cells. **(B)** Lung. Note the rapid decrease of radioactivity in dogs injected with myogenic stem cells, to reach values below 10% 7 days post-injection. (**C)** Liver. Note the high value observed in the dog injected with ^111^In-oxine-labelled leukocytes and the stable levels, around 20%, observed in the dog injected with ^111^In-oxine. A marked increase in radioactive signal is seen in the two days after injection in the dogs injected with myogenic stem cells, particularly the GRMD dogs injected with MABs. (**D**) Contralateral limb. The Y axis scale has been modified to fit in the low values of radioactivity measured in this ROI. Initially the highest values were obtained in the dog injected with ^111^In-oxine due to a skeletal tropism. In the dogs injected with myogenic stem cells, the radioactivity increased during the first 24 hours after injection to decrease again thereafter. **(E)** Whole body. This graph illustrates the stability of the signal, i.e. the excretion of ^111^In. In the case of the ^111^In-oxine-labelled leukocytes, the decay-corrected signal remained stable, meaning that the radioactivity remained in the cells and was not excreted. Conversely, in the case of dogs injected with myogenic stem cells, ^111^In was markedly excreted, this excretion was also observed in the dog injected with ^111^In-oxine, suggesting prominent leakage of ^111^In from the myogenic stem cells.

Quantitatively, the decay-corrected activity of the injected limb relative to the initial whole-body activity remained higher in GRMD dogs injected with MABs compared to WT dogs (p = 0.07 at 24 and 48 hours) and GRMD dogs injected with myoblasts (Fig. 3A). However, this relative activity, initially representing half of the whole-body activity, decreased to 12.1% (SD 2.4%) one week after injection. This decrease was also seen in WT dogs injected with MABs and GRMD dogs injected with myoblasts.

The lung was the second main territory where the activity was concentrated after myogenic stem cells infusion (Fig. 3B). As observed in the injected limb, the activity measured in the lung decreased during the days following injection, with a rapid drop in the first 24 hours, to reach values of relative activity of approximately 5%; these values were comparable among the three groups of dogs injected with myogenic stem cells. Conversely, and consistently with the observations made on the images, there was a marked increase in the relative activity in the liver; this increase was observed in all dogs injected with myogenic stem cells (Fig. 3C), with a peak 48 hours after injection and relative activity values ranging from 29.4% (SD 6.4%) in WT dogs injected with MABs to 46.2% (SD 1.8%) in GRMD dogs injected with myoblasts. This suggests that there is a redistribution of radioactivity from the injected limb and the lung to the liver.

Several reports have described the ability of MABs to recirculate and migrate to distant muscle groups^22,32^, therefore we sought to address whether our ^111^In-MAB tracking method was able to detect such behaviour. In order to do this, the radioactivity present in the contralateral limb was measured; the results of this analysis showed that the relative activity remained low over time (<2.0% in myogenic stem cell-receiving dogs) (Fig. 3D). The highest activity in the contralateral limb was measured in the dog injected with ^111^In-oxine with a progressive clearance of the signal, probably reflecting bone fixation and release of the radioisotope. The dogs injected with myogenic stem cells showed, in all cases, a progressive increase of the relative signal in the contralateral limb followed by a decrease; peak levels were seen between 24 and 48 hours after injection. These results indicate that there is a transient redistribution of radioactivity to the contralateral limb and this redistribution follows roughly the same time course as the redistribution to the liver.

Finally, the decay-corrected whole-body radioactivity was assessed as an indicator of stability of cell-labelling (Fig. 3E). A stable cell-labelling would entail that the signal decrease observed over time in whole-body activity should be close to the normal decay of ^111^In. This was indeed the case for ^111^In–labelled leukocytes, for which the decay-corrected whole-body activity remained very stable over time with no significant excretion of the radioisotope, as expected and described elsewhere^37,38^. In marked contrast, the decay-corrected whole-body activity strongly decreased in the animals injected with myogenic stem cells, reaching mean levels of 64.1% (SD 8.4%) one week after MAB injection in GRMD dogs. This observation indicates that one third of the ^111^In had been excreted at this time-point. This profile of elimination was comparable to the one observed in the animal injected with ^111^In-oxine within the first 48 hours, showing that the radioactivity of the injected myogenic stem cells was not retained in the organism beyond the retention observed when the radioactivity was injected as a free isotope. These results suggest that either there is radioisotope leakage from myogenic stem cells and/or that the transplanted cells are massively dying after injection.

### Cell viability of labelled MABs and ^111^In release: *in vitro* studies

**Figure 4:**
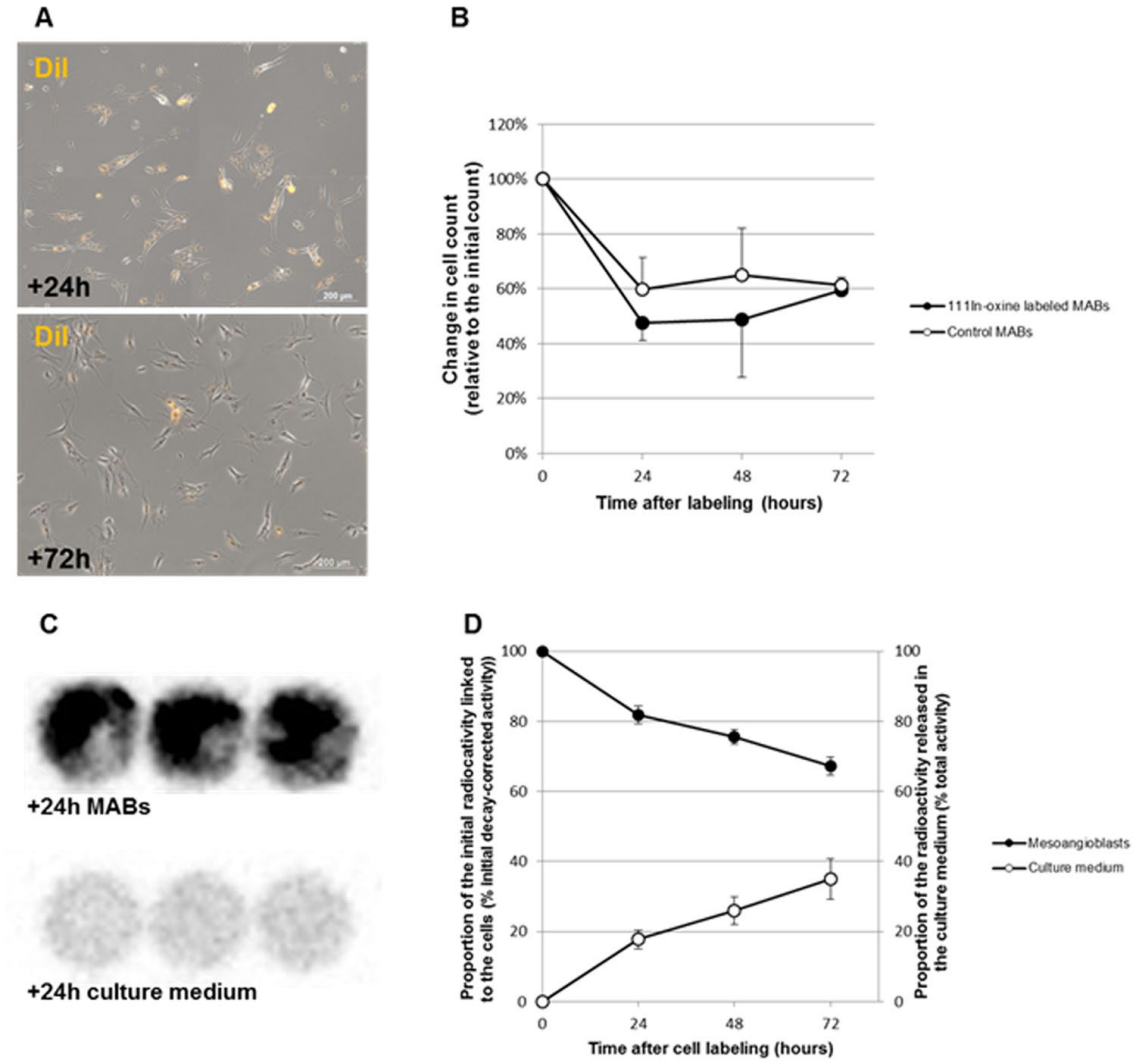
Cell viability, ^111^In release and myogenicity: *in vitro* experiments. (**A**) Merged DsRed-Brightfield pictures of DiI-^111^In-oxine-labelled MABs, 24 hours and 72 hours after labelling, showing similar cell densities in the dish. (**B**) Cell count 24, 48 and 72 hours after labelling, compared to the initial number of cells plated on day 0. Prominent cell loss and absence of proliferation was seen in both labelled MABs and unlabelled MABs to a slightly lesser extent. (**C**) Scintigraphic images of a 6-well plate 24 hours after labelling. The three upper wells contained labelled MABs and the three lower wells the corresponding culture medium. Both the labelled MABs and the culture media show radioactivity, indicating that there is ^111^In leakage from the cells. (**D**) Proportion of radioactivity remaining in the cells (black points) versus released in the culture medium (white points). This proportion confirms the release of a large proportion of ^111^In, probably due to cell mortality.

In order to better understand the results obtained *in vivo*, labelled MABs and similarly-processed unlabelled MABs were plated into dishes and counted 24, 48, and 72 hours after the labelling procedure (Fig. 4A). In both conditions, a striking decrease in cell number was observed in the first 24 hours, with levels ranging from 40.3% (SD 11.6%) decrease for unlabelled cells to a 52.4% (SD 6.4%) decrease in labelled cells. These results indicate that significant cell mortality was occurring. The underlying reason for this significant cell death was most likely related to cell manipulation *per se*, and to a lesser extent to cell labelling. A similar decrease in cell viability has previously been described in mesenchymal stem cells^10^. During the following 48 hours, the number of cells tended to stabilise in both experimental conditions, suggesting that there was a reduction in cell mortality but that it was not accompanied by an increase in proliferation (Fig. 4B). Several factors may be responsible for the effects observed, among them are sub-optimal cell culture conditions (20% O_2_ instead of the recommended 5%^39^) and cell senescence induced by the extensive expansion required for the injection and by peri-labelling procedures.

Gamma-camera imaging of cells and culture media revealed that radioactivity was present in the cells but it was also found in the culture medium (Fig. 4C). Quantitative analysis showed that the proportion of radioactivity remaining in the cells progressively decreased to reach 67.2% (SD 2.6%) three days after labelling, indicating that approximately one-third of the ^111^In was released to culture medium within this time span (Fig. 4D).

### *Ex vivo* tissue analysis and histological study

**Figure 5:**
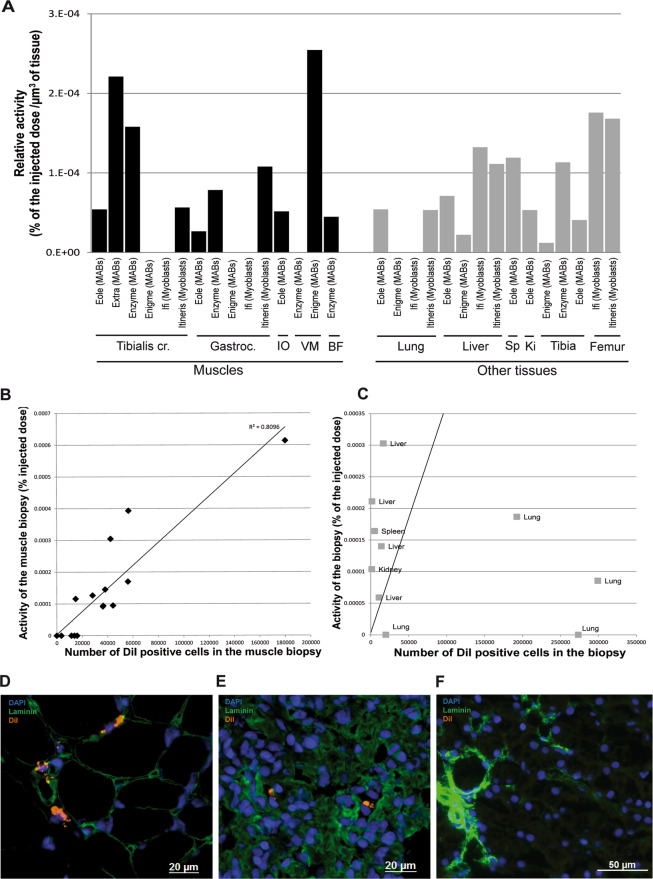
Correlation of imaging versus histology results. (**A**) Radioactivity of the muscle tissue samples from the injected limb (black bars in the histogram), and other tissue samples (grey bars), expressed as a percentage of the injected dose, and normalised by the volume of the sample. Each bar of the histogram represents a sample from one dog. (**B**) Correlation plot between the radioactivity of the muscle samples and the number of DiI^+^ cells. The results show that there is correlation between the images and radioactivity. (**C**) Correlation plot between the radioactivity of the samples from non-muscle tissues, and the number of DiI^+^ cells, showing no correlation. The regression straight line obtained for muscles was superimposed on this graph illustrating the fact that radioactivity in the liver was not associated with the presence of DiI^+^ cells, and that DiI^+^ cells in the lung did not correlate with the presence of radioactivity. In this latter tissue, the DiI^+^ signal is thus probably not arising from transplanted cells, but rather result from a transfer from dead cells to host cells. (**D**) Image from a muscle sample showing DiI^+^ cells (DiI in orange) (**E**) Image from a lung sample showing DiI^+^ cells (**F**) Image from a liver sample showing no DiI^+^ cell. Abbreviations: Tibialis cr.: *tibialis cranialis*; Gastroc.: *gastrocnemius*; IO: *interosseous*; VM: *vastus medius*; BF: *biceps femoris*; Sp: spleen: Ki: kidney.

After the last imaging session of the animals, different tissues were sampled and imaged using the gamma camera. Most of the muscles of the injected limb showed radioactive signal except for one of the myoblast-receiving dogs (“Ifi”) (Fig. 5A). However, the radioactivity measured was variable among muscles, even within the same animal. In one dog treated with labelled MABs (“Enigme”), the radioactivity was strong and restricted to the *vastus medius* muscle, most likely due to a selective injection. Among the non-muscle tissues, the lung was the tissue with the lowest radioactive signal, with levels that were almost undetectable. All the liver samples showed radioactive signals as well as the bone.

In order to perform a correlation between radioactivity levels and the number of cells, the tissue samples were frozen after imaging. The first step in this correlation was to detect, by qPCR, presence of the SRY gene. Presence of the SRY was used as a marker of stem-cell engraftment when male donor cells were transplanted into female receiver dogs. The number of male genomes was in most cases under the detection threshold; the SRY gene was only amplified in the *vastus medius*, from the dog “Enigme” (the most radioactive tissue sampled). In this case, even though the SRY gene could be detected, the amplification was observed in the 39.01^th^ cycle of the qPCR.

Thereafter, we changed the approach for the correlation study and used histological analysis to count the number of cells engrafted in the tissue rather that estimating this number by qPCR. This was performed by counting the number of DiI^+^ cells in a given sample and comparing this number to the activity measured in this sample. In muscle, the majority of the DiI^+^ cells (in animals treated with either myoblasts or MABs) were found in the lumen of the microvessels, as previously described for myoblasts^29^. The activity of the muscle samples from the injected limb substantially correlated (Pearson R = 0.90, p < 0.001) with the amount of DiI^+^ cells (Fig. 5B,D). However, the correlation was lost when other tissues were studied (Pearson R = −0.32, p = 0.36). The overall radioactivity of the lung underestimated the amount of DiI^+^ cells that were found to be numerous in most cases (Fig. 5C,E), whereas the radioactivity found in other tissues, particularly the liver, was high while very few DiI^+^ cells were found in the tissue (Fig. 5C,F). In conclusion, the radioactive signal reflected the presence of stem cells only in the injected muscles, but this correlation could not be extrapolated to the whole body, where the presence of radioactivity was no longer associated to the presence of labelled cells.

## Discussion

This study aimed to evaluate the reliability of a direct labelling method to track myogenic stem cells using scintigraphy in a large preclinical animal model of DMD. We used a widely known cell labelling method with ^111^In-oxine, which is easy to perform, and was in our hands efficient enough to yield satisfactory levels of myogenic stem cell labelling.

Radioactive cells could be tracked *in vivo* after intra-arterial delivery revealing a very specific immediate biodistribution pattern following infusion. A capillary barrier effect was obvious, with labelled cells trapped by the two first capillary filters they reached; firstly the capillary system of the injected hindlimb and thereafter the capillary system of the lung. This specific biodistribution pattern could be mediated by the adhesion properties of these cells to the endothelium. Alternatively, the large size of the myogenic stem cells (25–35 µm versus 7–20 µm e.g. for leukocytes) could account for mechanical trapping by microvessels, as previously described in non-human primates following intra-arterial injection of myoblasts^29^. This first capillary barrier effect emphasises the importance of using the intra-arterial administration route to optimise tissue targeting. As a consequence, a whole-body treatment of a DMD patient with such a cell therapy product would necessitate several sites of injection, including coronary arteries to target the cardiac muscle, and phrenic artery to target the diaphragm. The ^111^In-oxine labelling method could be used in further preclinical studies to assess the ability of these muscles to trap stem cells, relative to limb muscles.

Despite this immediate cell trapping in the infused limb in dogs injected with either myoblasts or MABs, differences were seen between the two cell types. Myoblasts, which are known to have poor migratory capacity to reach the muscle from the vascular compartment^29^, were retained to a lesser extent in the injected hindlimb than MABs. This underlines the fact that ^111^In-oxine based cell tracking is able to detect different cell behaviours and could be helpful in comparing the immediate biodistribution of different cell therapy products for degenerative muscle diseases. ^111^In-oxine based cell tracking using scintigraphy also suggested that more radioactivity was retained in the GRMD injected hindlimbs, than in the hindlimb of WT animals. One of the features that makes the dystrophic muscle tissue attractive for myogenic stem cell based treatments is that myogenic stem cells tend to migrate towards injured muscle by chemo-attraction mediated by cytokines released by the injured tissue^22,31^. This mechanism, well identified in mouse models, could be responsible for the differences observed between GRMD and WT dogs in the present study. However, the striking difference, detected as early as 1 hour after injection, could also be attributed to differences in microvessel architecture between the WT and the GRMD dogs. It has been described that microvessel density decreases with age in dogs^40^; in this study the WT dogs used were young adults, while the GRMD dogs were puppies, therefore it cannot be excluded that the difference in age could be partially responsible for the differences observed in cell trapping.

This method also proved able to describe differences in the cell homing between muscle groups and between dogs (Fig. S2). This is an interesting point because such images could contribute to the understanding of heterogeneous results among animals for a given muscle, as already observed in our initial study on MAB transplantation^32^. These differences are probably related to individual differences in vascularisation and to the site of intravascular infusion. Images acquired immediately after cell infusion could thus be helpful to map the initial cell homing in the injected limb and orientate subsequent muscle biopsies and functional evaluation.

*In vitro* experiments showed that there is significant cell mortality during the hours and days following cell labelling and transplantation, and that this mortality is associated with release of the radioisotope. The quantitative data obtained using *in vivo* scintigraphy also suggest that ^111^In is massively excreted following administration of labelled cells, at a rate equivalent to the one measured when ^111^In-oxine is injected as a free isotope. These results contrast with the stability of the labelling of leukocytes, that retained nearly 100% of the ^111^In as described previously^37^. The cell mortality observed occurred even when cells were unlabelled indicating that the reduction in cell viability is in part resulting from cell manipulation. However we cannot rule out that the toxicity of ^111^In could also be a contributing factor to cell death, as already described for some other cell types, in a cell-type dependent manner^41–43^. Despite the impact of the labelling procedure on cell viability, the surviving cells could have preserved some of their differentiation capacity, since we observed fusion of labelled MABs co-cultured with C2C12 in one experiment (data not shown).This question would need to be more deeply addressed, as well as the one of the impact of the labelling on migratory ability of MABs. This is another important biological property of these cells which should be addressed in further experiments, all the more so, since ^111^In-induced modifications have been described in granulocytes^44^.

The high mortality rate of myogenic stem cells observed during the first hours after labelling was responsible for the ^111^In release seen *in vitro*, and the marked excretion of radioactivity *in vivo* in the days following transplantation. Immediately after the injection of ^111^In-oxine, most of the radioactivity was detected in the liver. In addition, it is well described that ^111^In-oxine binds to transferrin in blood and is subsequently directed to the liver and the bone marrow^45^. In the case of myogenic stem cells, the initial radioactivity present in the injected limb and the lung progressively decreased while increasing in the liver; most samples of this latter tissue showed significant amount of radioactivity 7 days post-injection. Analysis of the biodistribution of DiI^+^ cells showed, however, that this radioactivity was not associated with the presence of DiI^+^ cells indicating that the uptake of ^111^In in the liver was mainly due to ^111^In released from dead cells. The same probably holds true for many other tissues, such as the bone marrow in which radioactive signal was detected, probably accounting for the apparent transient recirculation to the contralateral limb. This non-specific signal makes interpretation and quantification of the images in the days following injection difficult and precludes any direct translation of image analysis into reliable information on cell biodistribution mapping. Even if only a new correlation study at 24 and 48 hours could ascertain this, it is highly probable that this non-specific signal begins to scramble images very early on after injection, when cells start to die and release ^111^In. Of the many studies using direct ^111^In labelling-based cell tracking, only a few address ^111^In release and the subsequent non-specific signal. Our study clearly shows that the data obtained from these images, and especially those showing liver uptake, should be interpreted with caution.

More generally, one could speculate that any direct labelling method could potentially result in unspecific signals as the one observed in our study. This was shown to be the case with SPIO labelled cells for MRI tracking; the persistence of the signal in the absence of cells was demonstrated, and resulted from phagocytosis of the labelling agent by macrophages^16,46^. In the same way, in the present study, we cannot exclude that some of the DiI^+^ cells visualised are not transplanted cells, but result from a transfer of the dye to host-cells. This is likely the case in the lung, where the injected cells probably died rapidly releasing their ^111^In content in the vasculature, while the fluorescent dye was taken up by adjacent cells, as described by others^47^.

The main objective of this study was to assess whether ^111^In labelling of stem cells and *in vivo* tracking by scintigraphy could be a reliable tool to monitor myogenic stem cell biodistribution. We provided the first depiction of immediate MABs homing in a large animal model of DMD. We demonstrated that immediate scintigraphy and SPECT/CT acquisitions can be useful to monitor cell trapping in the injected limb, cell distribution in the different muscle groups and compare different cell types. However, the non-specific signal arising from ^111^In released by the labelled cells as well as the potential toxicity of the radioisotope make this method inconvenient to monitor stem cell biodistribution and recirculation in the hours/days following transplantation. Since we clearly demonstrated that the use of direct labelling methods should be limited to immediate biodistribution studies, an alternative direct labelling method would be the use of ^99m^Tc-HMPAO that has a short half-life (6 hours) allowing for a biodistribution snapshot immediately after injection, with a more favourable dosimetry than ^111^In and reduced housing constraints for dogs^7,11^. More ideally, indirect labelling methods through the insertion of a reporter gene, such as the NIS (Sodium Iodide Symporter), in the genome of cells could represent an attractive alternative to solve the problem of non-specific signal inherent to direct labelling methods and exemplified in the present study^17,18^. Moreover, it would allow long-term cell tracking studies, using NIS-compatible isotope such as ^99m^Tc^48^. This approach would thus open the way for ambitious preclinical studies combining cell tracking and functional evaluation.

## Methods

### Animals

All procedures were approved by the Ethical Committee of EnvA, ANSES and UPEC under the approval number 20/12/12-20 and were performed in accordance with the relevant guidelines and regulations.

A total of 12 dogs were enrolled in this study (Table 1), 7 GRMD dogs and 5 healthy adult beagle dogs. The latter were used as healthy controls for ^111^In-labelled MABs injections (n = 3), as reference for the biodistribution of ^111^In-oxine alone (n = 1) and of ^111^In-labelled leukocytes (n = 1). Healthy Beagles were chosen rather than GRMD healthy littermates because their size is closer to that of young GRMD dogs. At this age, healthy Golden Retrievers are much taller than GRMD littermates, and this would have impaired homogeneity in image acquisitions and analysis.

Dogs grafted with allogenic stem cells received immunosuppressive treatment that was initiated 3 weeks prior injection: prednisolone (2 mg/kg/d) and cyclosporin A (initial dose 20 mg/kg/d, adjusted to maintain trough levels of cyclosporinemia between 300 and 450 ng/ml), administered twice daily.

### Cell culture and labelling

Two types of myogenic stem cells were used: canine MABs and myoblasts both obtained from muscle biopsies sampled from healthy male dogs, according to previously described procedures^39,49^.

The day of the injection, cells were trypsinised and maintained in suspension in culture medium with 5% serum obtained from the recipient dog until labelling. A total of 40.10^6^ cells were labelled to be injected in each dog.

For the leukocyte experiment, a total of 115.10^6^ leukocytes isolated from a blood sample from the dog to be injected were used.

Prior to this study, preliminary *in vitro* experiments were performed to optimise labelling conditions, allowing for good post-labelling cell viability and labelling efficiency. The best conditions were used in the present study, as follows. Cells in suspension were first labelled with a fluorescent dye, 2.5 µM CM-DiI (Molecular Probes) during 15 minutes at room temperature. Thereafter, cells were centrifuged and resuspended at a density of 10.10^6^ cells/mL culture medium. A dose of 185 MBq of ^111^In-oxine was prepared and the pH stabilised at 7 using a Tris buffer solution; this solution was subsequently added to the cell suspension and incubated for a period of 15 minutes. Following incubation, 500 µl of serum obtained from the recipient dog were added, the cell suspension was centrifuged and the cells resuspended in 0.9% NaCl with 12.5 UI/ml heparin (1.10^6^ cells/mL). The labelling efficiency was calculated by dividing the measured radioactivity of the cells by the total radioactivity obtained by adding the radioactivity of cells, of the supernatant and of the vial used for labelling. The 40 mL cell suspension was transferred into syringes placed in lead holders and injections were performed almost immediately except in two cases that received the injection approximately 1 hour after the end of the labelling procedure (“Eole” and “Extra”). Following injection, the immediate cell viability was evaluated using the Trypan blue exclusion test.

In order to better understand the images obtained on dogs, a sample of the MABs prepared for two of the dogs (‘Enigme’ and ‘Enzyme’) were plated back into dishes to evaluate viability and growth capacity after labelling. MABs from the same batches, treated using the same procedures as the injected cells but without DiI or ^111^In-oxine labelling served as controls. Gamma-camera images of cells were obtained 24, 48 and 72 hours after labelling. The culture medium was imaged separately at the same time-points. Immediately after imaging, cells were trypsinised and counted.

### Intra-arterial injection

Given the fact that previous studies on MABs transplantation in preclinical models and DMD patients used intra-arterial delivery to perfuse limbs, we decided to keep the same administration route for this study. Dogs were anaesthetised using an intravenous injection of propofol (6.5 mg/kg), followed by inhalation of isoflurane (2–3%) in 100% O_2_. One of the femoral arteries was mounted on two vessel loops and 22 G catheter (Vasocan Braunüle B.Braun) was inserted in the artery between the two loops. Forty mL of labelled cell suspension were then manually injected over a period of 5 minutes through an extension line connected to the catheter.

### Imaging

Imaging was performed on a GE Healthcare Infinia II 3/8 Hawkeye Hybrid NM/CT, with medium energy general purpose collimators and two energy windows peaked at 171 and 245 keV.

On day 0, a dynamic imaging sequence lasting 30 minutes was acquired during and after injection using a 64 × 64 matrix. For this sequence, the dog was placed transversally to the bed, in dorsal recumbency with limbs in flexion to allow for whole-body imaging.

After suturing the surgical site, the dog was placed longitudinally to the bed, in dorsal recumbency with extended limbs and a first static planar whole-body acquisition was achieved around 1 hour after injection using a 256 × 256 matrix. A SPECT/CT sequence on the hindlimbs was thereafter acquired using a 128 × 128 matrix, a pixel size of 4.42 × 4.42 mm, and 60 30-second-long projections on dual cameras.

Static planar whole-body acquisitions and SPECT/CT on the hindlimbs were retaken approximately 2 hours after injection, on days 1 and 2. The same imaging protocol was performed on day 7 on the GRMD dogs (n = 5 MABs and 2 myoblasts), and on one WT MAB-receiver.

For imaging of cell cultures and biopsies, a static acquisition sequence lasting 5 minutes was obtained.

### Image analysis

The quantitative analysis was performed on static whole-body planar acquisitions, in the Xeleris (GE Healthcare) workstation. Regions of interest (ROIs) were drawn around the injected limb, the contralateral limb, the lung, the liver and the kidneys and when visible, the spleen and the heart. The area of the ROIs was kept constant from an imaging timepoint to another. The activity of each ROI was decay-corrected and normalised by the whole-body activity measured on the first static acquisition.

### Histological analysis

After the last acquisition 7 days after injection, two GRMD dogs grafted with myoblasts, and three GRMD grafted with MABs were euthanised. Two MAB-grafted GRMD dogs underwent a *tibialis cranialis* muscle biopsy. Several muscle and non-muscle tissue samples were taken from the euthanised dogs and imaged with the gamma-camera. ROIs were drawn on the images and the activity of each sample was quantified by subtracting the area-corrected noise value. On the same acquisition, a tube containing a known dose of ^111^In on the day of injection was also imaged; the activity of each sample was expressed as a percentage of the total dose of ^111^In injected to the dog after decay-correction and normalisation by the sample volume.

Immediately after imaging, the tissue samples were snap frozen in isopentane cooled in liquid nitrogen. The frozen samples were cut entirely and the volume of each sample was calculated by measuring the slice surface every 1500 µm, and calculating the sample height by counting the number and thickness of cryostat sections. On one slice every 1500 µm an anti-laminin immunofluorescence assay (Sigma, rabbit polyclonal, 1/100) was performed to visualise tissue structures. Visualisation was performed by scanning the complete slice. The number of DiI-labelled cells was manually counted on each slice and the results obtained were extrapolated to the whole sample taking into account the cell density on each slice and the volume of the sample. The number of cells per sample was thereafter compared with the activity obtained for each sample.

### qPCR SRY

After DNA extraction from the tissues sampled from the female receivers, qPCR was performed in a LightCycler 96 System (Roche) using SYBR-Green Master Mix (Thermo Scientific). Duplicates of DNA were tested for SRY (male donor); NROB and RPS19 were used as housekeeping genes. Primers sequences 5′-3′: RPS19- Forward: CCTTCCTCAAAAAGTCTGGG, RPS19-Reverse: GTTCTCATCGTAGGGAGCAAG; NROB1-Forward: CCAGCAGGAGCACTGTCTTT, NROB1-Reverse: TGGGATGGAGTCAGGGTGAA; SRY-Forward: CTCGCGATCAAAGGCGCAAG, SRY-Reverse: TTTCGGCTTCTGTAAGCATTTTCC.

### Statistical analysis

A Mann-Whitney test was used to compare healthy versus GRMD dogs transplanted with MABs. A Spearman rank test was performed to assess the correlation between cell viability and immediate relative radioactivity in the injected limb. A Pearson’s coefficient of correlation was calculated in order to study the correlation between the amount of radioactivity and the number of DiI^+^ cells present in the tissue samples. A p-value ≤ 0.05 was considered statistically significant.

## Supplementary information

Supplementary Information.

Supplementary Information 2.

Supplementary Information 3.

## Data Availability

The datasets used and/or analysed during the current study are available from the corresponding authors on reasonable request.
