# Supplementary material for: In vivo stem cell tracking using scintigraphy in a canine model of DMD
Source: Sci Rep. 2020 Jun 30;10:10681. doi: 10.1038/s41598-020-66388-w (PMC7327062; doi:10.1038/s41598-020-66388-w)
Supplement: Supplementary file 1 — Supplementary Information. [file 41598_2020_66388_MOESM1_ESM.docx]

**Title:** *In vivo* stem cell tracking using scintigraphy in a canine model of DMD.

**Author list**: Inès Barthélémy^1†*^, Jean-Laurent Thibaud^1,2†^, Pauline de Fornel^2^, Marco Cassano^3,4^, Isabel Punzón^1^, David Mauduit^1^, Jean-Thomas Vilquin^5^, Patrick Devauchelle^2^, Maurilio Sampaolesi^3^, Stéphane Blot^1*^.

**Figure S1: Movie of a dynamic acquisition during and immediately after injection**

**Figure S2: Compared SPECT/CT imaging on two GRMD dogs injected with MABs**

Landmarks have been drawn on planar views (on the left and on the right) to schematize the location of the axial views in SPECT/CT (images in the middle of the figure, upper images = thighs, lower images = legs). A: Images from the dog ‘Enigme’ showing a prominent ^111^In uptake in the *vastus medius* muscle (asterisk) due to a probably selective arterial injection, and almost no radioactivity in other muscles, especially in the leg. B: Images from the dog ‘Enzyme’, showing a more disseminated signal among muscle groups, downstream the injection site, with a preferential localization to the medial part of the thigh (hash) and the cranial part of the leg (*tibialis cranialis* muscle, circle).

**Supplemental methods**

Primers sequences (5’-3’):

RPS19- Forward: CCTTCCTCAAAAAGTCTGGG

RPS19-Reverse: GTTCTCATCGTAGGGAGCAAG

NROB1-Forward: CCAGCAGGAGCACTGTCTTT

NROB1-Reverse: TGGGATGGAGTCAGGGTGAA

SRY-Forward: CTCGCGATCAAAGGCGCAAG

SRY-Reverse: TTTCGGCTTCTGTAAGCATTTTCC
