# Supplementary figures and images for: In vivo stem cell tracking using scintigraphy in a canine model of DMD
Source: Sci Rep. 2020 Jun 30;10:10681. doi: 10.1038/s41598-020-66388-w (PMC7327062; doi:10.1038/s41598-020-66388-w)

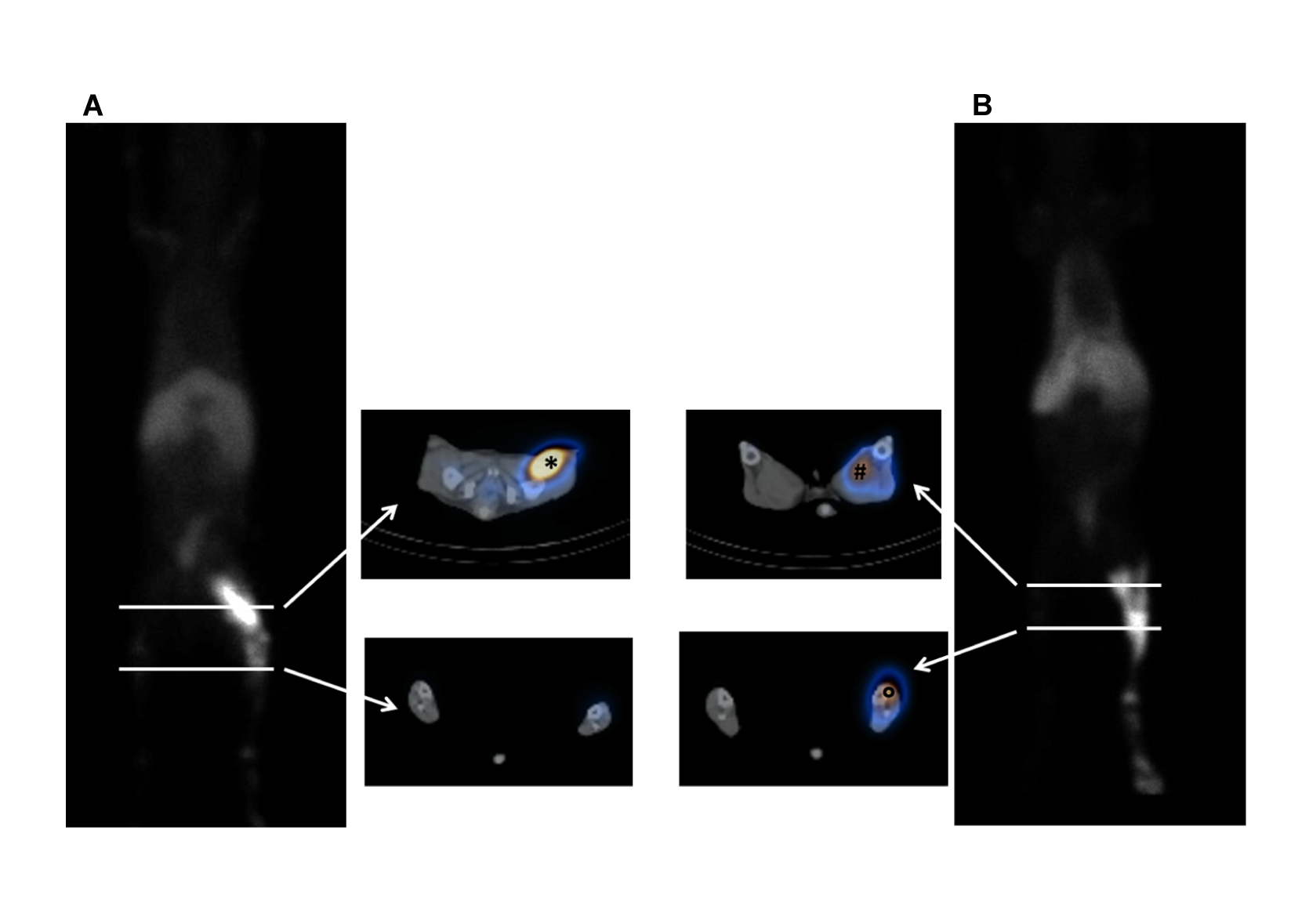

Supplement: Supplementary file 3 — Supplementary Information 3. [file 41598_2020_66388_MOESM3_ESM.tif]
